# Olfactory Characterization and Training in Older Adults: Protocol Study

**DOI:** 10.3389/fnagi.2021.757081

**Published:** 2021-11-16

**Authors:** Fabíola Zambom-Ferraresi, Fabricio Zambom-Ferraresi, Joaquín Fernández-Irigoyen, Mercedes Lachén-Montes, Paz Cartas-Cejudo, Juan José Lasarte, Noelia Casares, Secundino Fernández, Bernardo Abel Cedeño-Veloz, Enrique Maraví-Aznar, Maria Itziar Uzcanga-Lacabe, Arkaitz Galbete, Enrique Santamaría, Nicolás Martínez-Velilla

**Affiliations:** ^1^Geriatric Unit, Navarrabiomed, Hospital Universitario de Navarra (HUN), Universidad Pública de Navarra (UPNA), Instituto de Investigación Sanitaria de Navarra (IdisNa), Pamplona, Spain; ^2^Clinical Neuroproteomics Unit, Navarrabiomed, Hospital Universitario de Navarra (HUN), Universidad Pública de Navarra (UPNA), Instituto de Investigación Sanitaria de Navarra (IdisNa), Pamplona, Spain; ^3^Immunology and Immunotherapy Program, Centro de Investigación Médica Aplicada, Instituto de Investigación Sanitaria de Navarra (IdisNa), Universidad de Navarra, Pamplona, Spain; ^4^Department of Otolaryngology, Clínica Universidad de Navarra, Facultad de Medicina, Universidad de Navarra, Pamplona, Spain; ^5^Department of Geriatrics, Hospital Universitario de Navarra (HUN), Pamplona, Spain; ^6^Department of Otolaryngology, Hospital Universitario de Navarra (HUN), Pamplona, Spain; ^7^Department of Statistics, Computer Science and Mathematics, Universidad Pública de Navarra (UPNA), Pamplona, Spain

**Keywords:** smell sense, olfactory dysfunction, immune fitness, odor training, geriatric, neurodegenerative disease, olfactory epithelium, proteome profile

## Abstract

The aim of this article is to present the research protocol for a prospective cohort study that will assess the olfactory function and the effect of an intervention based on olfactory training in healthy very old adults (≥75 years old). A convenience sample of 180 older people (50% female) will be recruited in three different environments: hospitalized control group (CH) with stable acute illness (*n* = 60); ambulatory control group (CA) of community-based living (*n* = 60); and an experimental odor training group (EOT) from nursing homes (*n* = 60). The odor training (OT) intervention will last 12 weeks. All the volunteers will be assessed at baseline; CA and EOT groups will also be assessed after 12 weeks. The primary end point will be change in olfactory capacity from baseline to 12 weeks period of intervention or control. The intervention effects will be assessed with the overall score achieved in Sniffin Sticks Test (SST) – Threshold, Discrimination, and Identification (TDI) extended version. Secondary end points will be changes in cognitive tasks, quality of life, mood, immune status, and functional capacity. All these measurements will be complemented with an immune fitness characterization and a deep proteome profiling of the olfactory epithelium (OE) cultured *ex vivo*. The current study will provide additional evidence to support the implementation of olfactory precision medicine and the development of immunomodulatory nasal therapies based on non-invasive procedures. The proposed intervention will also intend to increase the knowledge about the olfactory function in very elderly people, improve function and quality of life, and promote the recovery of the health.

## Introduction

Europe is aging. In 2050, more than 28% of the population will be over the age of 65 (United Nations, 2019). The continuous declining of birth rates and the increasing of life expectancy are converting European regions into increasingly aging area. Neurodegenerative diseases (ND) are a significant portion of neurological disorders and these already account for 1/3 of the cost of illness in Europe furthermore social and economic burden will continue to increase as the population ages (Deuschl et al., 2020). Addressing the human and economic challenge of aging and neurodegenerative diseases requires clinical and research tools that facilitate precision medicine. We need disruptive approaches that generate innovative tools for early detection and diagnosis of ND and non-invasive interventions to slow and/or stop its progression in women and men, highlighting their differences if any, and incorporating a gender perspective in the solutions.

The olfactory function is responsible for detecting and processing odors, and is one of the oldest and most important senses for living organisms (Delgado-Losada et al., 2020). The sense of smell provides important information about the air we breathe, the foods we eat and alert us to dangers in the environment (Schiffman, 1997; Hadley et al., 2004; Schriever et al., 2014). Beyond the risks that the smell loss may entail for older people, moreover the consequent reduction in quality of life, the olfactory dysfunction may be an important early indicator of ND such as Parkinson’s and Alzheimer’s (Djordjevic et al., 2008; Haehner et al., 2013; Knudsen et al., 2015; Doty, 2017; Marin et al., 2018; Kondo et al., 2020), as well as COVID-19 (Moein et al., 2020; Sedaghat et al., 2020). In addition, olfactory system dysfunctions have been found in other neurological disorders such us schizophrenia, or depression (Sanna et al., 2021) ultimately related to alterations of the immune system, suggesting that olfactory system abnormalities may be also associated with the immune system (reviewed in Strous and Shoenfeld, 2006). During the last years, olfactory proteomics has been postulated as a powerful approach to characterize global proteome dynamics in order to unravel the modulation of cell-signaling networks during odor processing (Lachén-Montes et al., 2016), as well as during the neurodegenerative process (Lachén-Montes et al., 2017, 2019a,b, 2020a).

In the literature, there is a full consensus stating that sense of smell gradually decreases with age, especially after the age of 60 (Doty et al., 1984; Murphy et al., 2002; Doty et al., 2011; Mullol et al., 2012; Hummel, 2014; Sorokowska et al., 2015; Masala et al., 2018; Doty, 2019; Kondo et al., 2020). In relation to sex differences, by one side Doty et al. (1984) have observed that women outperformed men at all ages in healthy subjects, and a recent meta-analysis has confirmed the female olfactory superiority, although authors emphasized the effect sizes observed were notably small (Sorokowski et al., 2019). By other side, when comparing healthy control subjects with Parkinson’s disease patients, new findings (Melis et al., 2019; Solla et al., 2020) have remarked the importance of sex effect analysis. Regarding smoking, there is no consensus indicating that it accelerates olfactory dysfunction (Vennemann et al., 2008; Fjaeldstad et al., 2021). However, there is evidence that current smoking, but not former smoking, was associated with posttraumatic olfactory loss; namely, a history of smoking was not associated to impairment of olfactory function (Vennemann et al., 2008). Related to lifestyle and behavior factors, the regular exercise was associated with a lower 10-year cumulative incidence of olfactory impairment; in addition, the association with exercise was more robust among those who exercised three or more times per week than among those who only exercised 1–2 times per week (Schubert et al., 2013).

Training and repeated exposure to odorants leads to enhanced olfactory sensitivity (Al Aïn et al., 2019). The olfactory or odor training (OT) is a non-invasive technique with no significant side effects. Previous studies have proven the usefulness of OT in handling olfactory deficits with various etiologies (Hummel et al., 2009; Patel, 2016; Pekala et al., 2016; Al Aïn et al., 2019). The OT may be considered a simple protocol, which has proven efficacy in some patients with olfactory dysfunction. Despite increasing acceptance as an appropriate intervention, questions related to both efficacy and mechanism of action have persisted (Hummel et al., 2009; Turner, 2020).

In non-expert individuals with a normal sense of smell, a short-term OT improves olfactory performance (Dalton et al., 2002), and repeated exposure to an odorant enhances odor detection (Engen, 1960; Doty et al., 1981; Rabin and Cain, 1986; Dalton et al., 2002). Along the same lines, a few seconds of OT daily is considered as a behavioral therapy in patients with olfactory dysfunction (Abolmaali et al., 2002; Mueller et al., 2005; Rombaux et al., 2006; Hummel et al., 2009; Fleiner et al., 2012; Haehner et al., 2013; Konstantinidis et al., 2013; Damm et al., 2014; Schriever et al., 2014; Al Aïn et al., 2019). There is evidence that OT may increase olfactory sensitivity in Parkinson’s disease (Haehner et al., 2013). Notably, it has been also shown in animal models that odor training may have an impact on their cognitive functions (Lasarte-Cia et al., 2018). Finally, the evidence about OT in healthy older population and its efficacy is scarce (Schriever et al., 2014; Birte-Antina et al., 2018). Table 1 shows a brief survey of OT protocols found in the literature.

**TABLE 1:** Review of odor training protocols.

References	Study type Sample etiology	Sample (Mean age ± SD)	Test	Methods	Results
Al Aïn et al. (2019)	Randomized controlled trial Healthy population	*N* = 36 (24; 18–35) 58% ♀ Exp1 (OT) = 12 Exp2 (VCT) = 12 Con = 12	SST; UPSIT	Exp1(OT) = 6 weeks 1/day Intensive training (20’) Exp2(VCT) = visual control training Con = no training	Intensive OT can improve olfactory function. Improvement is associated with changes in the structure of olfactory processing areas of the brain.
Birte-Antina et al., 2018	Controlled, unblinded, longitudinal study Older people with normal olfactory function (>27/28 points in TDI score)	*N* = 91 78% ♀ 50–84 (61.1, ± 8.7) Exp(OT) = 60 Con = 31	SST	Exp(OT) = 20 weeks, 2/day 4 odors Con = Sudoku tasks	Positive effect of OT in older people on olfactory function, which extended to general mood and depressive symptoms. For cognitive function, a positive effect on verbal fluency was observed, but no effect on short-term memory or attentiveness.
Fornazieri et al. (2019)	Prospective Postinfectious, posttraumatic, or idiopathic olfactory loss	*N* = 25 68% ♀ (22–82 y) Exp1(classical training) = 12 Exp2(modified training) = 13	UPSIT	12- and 24-weeks testing 2/day for 24 months (10’ each) Exp1 = 4 odors Exp2 = 8 commercial products	Adherence rate of the patients after 3 months was 88% and after 6 months was 56%.
Gudziol et al. (2006)	Longitudinal Impairment of olfactory sensitivity (hyposmia or functionally anosmia)	*N* = 83 58% ♀ 12–84 y (58.2 ± 11.8)	SST	No intervention, olfactory testing was performing in two occasions (mean interval 136 days (7 days – 6.7 years)	Logistic regression showed that more than 60% of the subjects reported an improvement of olfactory sensitivity when the TDI score increased by 5.5 points. There is a statistically significant relation between measured and perceived improvement of olfactory function in patients who first presented with the diagnosis of anosmia or hyposmia.
Haehner et al. (2013)	Prospective, controlled nonblinded study Parkinson disease	*N* = 70 35% ♀ Exp(OT) = 35 (63.1 ± 8.3) Con = 35 (61.5 ± 9.5)	SST; threshold tests for the odors used in the training process	Exp(OT) = 12 weeks 2/day 4 odors (10” each) Con = no training	Significant increase in Exp group; and no changes in Con Group
Hummel et al. (2009)	Prospective Patients with olfactory dysfunction	*N* = 56 59% ♀ Exp(OT) = 40 (56 ± 11) Con = 16 (62.3 ± 13.4)	SST; threshold tests for the odors used in the training process	Exp(OT) = 12 weeks 2/day 4 odors (10” each) Con = no training	Positive significant increase in Exp group; and no changes in Con Group
Pekala et al. (2016)	Systematic review Olfactory loss Multiple etiologies		SST (6 out 10)		Statistically significant improvement (the mean difference in post treatment TDI score between groups was < 4 points.
Schriever et al. (2014)	Prospective Older people	*N* = 91 70% ♀ 55–96 (81 ± 8.6) Exp(OT) = 23 Con = 48	Odor identification and PEA threshold (SST)	Exp = 12 weeks 2/day 4 odors (30” each) Con = no training	No significant increase in olfactory function was observed in the training group.

*Exp, experimental group; Con, control group; SST, sniffin sticks test; TDI, threshold, discrimination and identification; UPSIT, university of Pennsylvania smell identification test; y, years old.*

Although there are important findings showing that intensive OT can improve olfactory function and in addition, this improvement is associated with changes in the structure of olfactory processing areas of the brain (Al Aïn et al., 2019). More research is needed to establish the optimal protocols i.e., quantity of odorants, training duration, associated-molecular events, and the target patient population most likely to improve using this technique (Patel, 2016). Therefore, the aim of this pilot study is to examine the effects of a well-controlled and semi supervised 12-weeks OT on olfactory function, OT-associated OE proteostatic changes, cognitive, immune and functional status, and quality of life in very elderly adults.

## Materials and Methods

### Study Design and Participants

The sample is a convenience sample and is composed by three groups (*N* = 180, 50% females). Sixty hospitalized patients with acute illness (hospitalized control group – CH), sixty ambulatory patients from the community (ambulatory control group – CA) and, sixty from nursing home – the experimental odor training group (EOT). The study follow the principles of the Declaration of Helsinki (World Medical Association, 2013) and was approved by the Complejo Hospitalario de Navarra Clinical Research Ethics Committee on October 21, 2020 (PI_2020/113). All volunteers or their legal representatives will be asked to sign a written informed consent form, also approved in advance by the ethics committee. There will be no financial compensation.

Following the literature procedures (Delgado-Losada et al., 2020) all the volunteers will be previously evaluated by an expert otolaryngologist including anamnesis and nasal endoscopic examination to exclude the possibility of an otorhinolaryngology alteration or pathology that may modify the results of the olfactory test. Therefore, sinusitis, prior nasal surgery, nasal polyps, presence of nasal congestion at the moment of test administration or recent upper respiratory tract infection within two weeks, medication intake with repercussion in olfactory performance or to have reported COVID-19 compatible smell symptomatology are exclusion criteria.

The inclusion criteria are: (i) ≥ 75 years old; (ii) compliance with testing procedure; (iii) Mini-Mental State Examination (MMSE) equal to or greater than 21 points (Pi et al., 1994; Ostrosky-Solís et al., 2000; Reyes-de-Beaman et al., 2004); (iv) Barthel index ≥ 60; (v) participants from acute hospitalized group must be clinically stable. The exclusion criteria are positive diagnosis of any kind of neurodegenerative disease and/or nasosinusal pathology (described before).

### Outcomes

The primary clinical outcome will be the overall score achieved in Sniffin Sticks Test (SST) extended version, which objectively assess the olfactory function through the Threshold, Discrimination, and Identification (TDI) score (Hummel et al., 1997). This olfactory test is comprised of three different components: olfactory threshold, odor discrimination, and odor identification. The olfactory threshold represents the level of detection of odors from low to high concentrations, the odor discrimination is the non-verbal distinction between different odors, and odor identification represents the ability to name or associate an odor. Using commercially available felt-tip pens, the odorants will be presented approximately 2 cm in front of both nostrils for 2 s (Hummel et al., 1997, 2007, 2009; Gudziol et al., 2006; Rumeau et al., 2016). Following the previous literature, we have established a 6 points improvement as a significant change (Hummel et al., 2009; Fleiner et al., 2012; Konstantinidis et al., 2013; Schriever et al., 2014; Pekala et al., 2016).

The **functional capacity** will be assessed with Short Physical Performance Battery (SPPB), which assessed balance, gait velocity, and leg strength (from 0 worst to 12 best scale) (Guralnik et al., 1994). In addition, to measure disability, the Barthel Index of independence during activities of daily living (ADLs) at the moment of assessment will be employed. This index ranges from 0 worst to 100 best (Mahoney and Barthel, 1965; van Bennekom et al., 1996). The magnitude of meaningful change (i.e., clinically significant) will be 1 point for SPPB (Perera et al., 2006) and 5 points for the Barthel Index (Shah et al., 1989; van Bennekom et al., 1996). Lastly, the changes in handgrip strength measured with a dynamometer (Takei 5401 Digital Dynamometer) will also be used to assess the functional capacity. The handgrip strength is a powerful predictor of disability (Rantanen et al., 1999), an indicator of overall muscle strength and is associated with a variety of clinically relevant health outcomes in aging adults (McGrath et al., 2018).

**Cognitive function** will be assessed by Mini-mental State Examination (MMSE) one of the most used instrument for screening cognitive impairment. The MMSE assesses domains of orientation, memory, attention, language, and visuospatial ability and is scored out of 30 points. Scores ≤ 23 points are indicative of likely cognitive decline (Folstein et al., 1975; Pi et al., 1994). The Trail Making Test part A (TMT-A) is a neuropsychological test that provides information on visual search, scanning, speed of processing, mental flexibility, and executive functions (Tombaugh, 2004). As the TMT-A is sensitive to a variety of neurological impairments and processes it will be employed to characterize the subjects and to observe changes in the groups that will be assessed at different point in time. The Symbol Digit Modalities Test (SDMT) is easily administered and sensitive to the presence of cerebral dysfunction (Smith, 1973). The score of this test is the number of correct substitutions completed within the time limit, with the maximum score of 110 (Smith, 2002). It is also sensitive to spontaneous recovery of brain functions and to improvements resulting from therapeutic treatments (Smith, 2002; Cancela et al., 2012).

**FIGURE 1:**
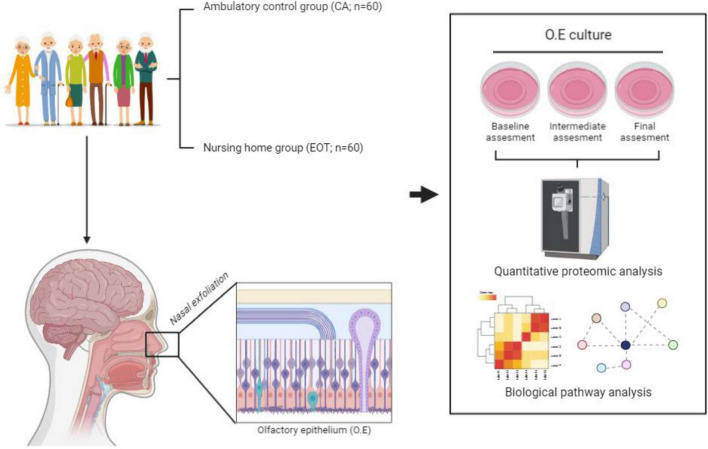
Olfactory proteomics workflow to be implemented in parallel with the EOT.

The **nasal exfoliation**, OE cell culture and subsequent quantitative proteomics will be performed in an ISO 9001:2015 quality environment as previously described (Lachén-Montes et al., 2020b; Barrera-Conde et al., 2021). The mass-spectrometry-based comparative protein analysis together with the corresponding bioinformatic phase (pathways and functional interactomes) will be specifically done in the EOT and CA environments (Figure 1).

To perform the **Immune characterization**, peripheral blood (PB) samples will be obtained from all groups at baseline and after 12 weeks from CA and EOT groups. EDTA blood collection tubes (Vacuette^®^, Greiner Bio-One) will be used. All PB samples will be processed according to the EuroFlow bulk lysis standard operating protocol (SOP) (available at www.EuroFlow.org). In brief, up to 2 ml of blood will be incubated with 50 ml of ammonium chloride (NH_4_Cl) at room temperature (RT) on a roller bank to lyse non-nucleated red blood cells. After a washing step, cells will be stained for 30 min in the dark with the following BD antibody combination: CD27, CD45RA, CD8, IgD, CD16, CD56, CD4, IgM, CD19, CD3, CD45, and either TCRαβ or preferably TCRγδ, wherein the following antibody pairs CD8/IgD, CD16/CD56, CD4/IgM, and CD19/TCRγδ or CD19/TCRαβ will be conjugated to the same fluorochrome (van Dongen et al., 2019). Finally, cells will be incubated for 10 min (in the dark; RT) with 2 ml of BD FACS™ Lysing Solution (BD Biosciences), washed once and re-suspended in 500 μl PBS prior to acquisition. Data acquisition will be using a BD FACSCanto II. For data analysis, the Infinicyt software (Cytognos, version 2.0) will be used. It provides a data integration and a multidimensional analysis of flow cytometry data.

The **mood status** will be assessed through the 15-item Yesavage Geriatric Depression Scale (GDS-VE), Spanish version, comprising ten affirmative and five negative items (from 0 best to 15 worst scale). The range between 0 and 5 suggest normal results and, scores greater than 5 indicates depression symptoms (Martínez et al., 2002).

**Quality of life** and the economic evaluation will be measured using the Spanish version of the EuroQol 5-Dimension 3-Level questionnaire (EQ5D-3L) (Badia et al., 1999; García-Gordillo et al., 2015). This instrument measures 5 dimensions of health status: mobility, self-care, usual activities, pain/discomfort, and anxiety/depression. In addition, the visual analog scale (VAS) will used to quantify perceived health (from 0, worst health state imaginable; to 100, best health state imaginable).

In order to have a complete phenotyping of subjects, sociodemographic variables (i.e., education level) will be studied. Finally, other end points included will be geriatrics syndromes, Cumulative Illness Rating Scale for Geriatrics (CIRS-G), polypharmacy and International Classification of Diseases (ICD-10). The analysis of all these variables will contribute to characterize the volunteers in the most comprehensive way. The full assessment will last in average 1 h and 20 min.

### Data Analysis

Demographic and outcome data will be summarized for the whole sample and for each group using mean and standard deviation or median and interquartile range for continuous variables and via frequencies and percentages for categorical ones. For the CH group only a descriptive characterization of the measured variables will be performed. In the CA and EOT groups, baseline data will be compared using t-test or Mann-Whitney U test for continuous variables and with chi-square test and Fisher test for categorical ones. For each group, within group pre-post differences will be computed using t-test for dependent samples or Wilcoxon rank sum test. Linear models will be computed in order to compare outcome data between groups, adjusting by baseline outcome data and also by the variables that showed baseline differences.

Further, the association between olfactory level measured with TDI score and marked OE protein modifications (based on fold-change), cognitive, neurological, functional, mood status and quality of life will be analyzed using Pearson or Spearman correlation coefficients. All significant, nearly significant, clinically relevant or potentially confounding variables will be considered in a multiple linear regression model with TDI as dependent variable. In this global model non-significant variables will be removed, which, when eliminated, do not change the estimators of the effect of the rest of the variables in the model. The biochemical analysis, the immune information of each subject and the data associated with pharmacological treatments, fragility, cognition, will also be correlated with olfactory level, with the aim of testing the precision olfactory medicine as a useful tool in the day-to-day context of the Geriatrics Unit. To exclude the effect of sex and age, correlations will be performed as partial correlations, including sex and age as covariates. The statistical analysis will be performed with SPSS (version 26; SPSS Inc., Chicago, IL, United States).

Sample size was calculated based on previous literature (Hummel et al., 2009) on the effects of OT on olfactory function, a medium effect size (*d* = 0.5) and a standard deviation of the outcome of *SD* = 0.8 were assumed (Birte-Antina et al., 2018).

### Intervention

#### Control

The control group will be composed by sixty ambulatory patients from the community (CA). This group will undergo a complete assessment at baseline and after 12 weeks. The volunteers of CA group will receive usual health care assistance; therefore they will not participate in any kind of olfactory training.

#### Training Protocol

**FIGURE 2:**
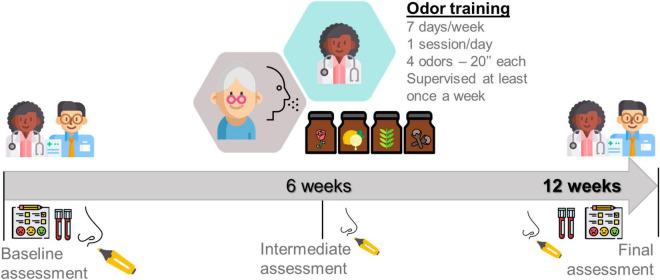
Odor training protocol (own elaboration).

The experimental odor training group (EOT) will be composed by sixty older adults from nursing home. This group will undergo a complete assessment at baseline and after 12 weeks. The training intervention will be performed over a period of 12 weeks and will involve patients exposing themselves once daily to 4 different scents of the four commonly used odors during 20 s each (phenyl ethyl alcohol (PEA): rose, eucalyptol: eucalyptus, citronellal: lemon, and eugenol: cloves). These four odorants were chosen to be representative of four odor categories that tried to identify/represent primary odors (Amoore, 1977; Haehner et al., 2013). These categories are flowery (e.g., rose), foul, fruity (e.g., lemon), aromatic (e.g., cloves), burnt, and resinous (e.g., eucalyptus). Training patients received four vial topaz (total volume 5 ml) with one of the four odors in each (1 ml each, soaked in cotton pads to prevent spilling). All vials will be labeled with the odor name. Patients will be asked to sniff the odors in the morning for approximately 20 s each. Participants will be asked to refrain from smoking, eating, or drinking (except water) during the hour prior to training and testing (Al Aïn et al., 2019). Figure 2 shows a summary outline of the proposed protocol.

Adherence to the OT intervention will be documented in an *Odor training diary* (P.R.O-V1.1E, 2020). By one side, some protocols in literature (Table 1) performed the training sessions twice a day or 12 weeks of OT (Hummel et al., 2009; Haehner et al., 2013; Schriever et al., 2014; Fornazieri et al., 2019). However, by other side, Schriever et al. (2014) and Fornazieri et al. (2019) have indicated that only half part of the sample strictly follow the protocol. In this regard, our main objective is to propose a protocol that can be followed in an easy and manageable way by our sample of very old adults. For this reason, we propose only one training session per day during a 12 weeks period. In the same way, seems important to highlight that at least once a week a researcher of our staff will supervise the training sessions and furthermore participants will receive a follow up call per week. Lastly, participants who do not experience a subjective perceived improvement in olfactory function tend to lose motivation, so there will be an intermediate smell test with SST at 6 weeks to assess objectively the evolution of performance.

## Expected Results

Following the overall evidence (Hummel et al., 2009; Pekala et al., 2016; Al Aïn et al., 2019) about OT, we expect EOT group increased their olfactory function measured by the TDI score, when compared to baseline assessment of both, intervention and control groups. The scarce evidence and its controversial results on elderly OT (Schriever et al., 2014; Birte-Antina et al., 2018) support the needed for further studies to confirm its efficacy.

Schriever et al. (2014) only have observed statistical significant difference between groups afterward the 12 weeks period, when training group have improved olfactory function and control group obtained lower results than the baseline. But the results of inter-group analysis (baseline and final assessment of OT group) are not significant. The authors (Schriever et al., 2014) suggest that there are several reasons for the lack of olfactory improvement and they are related with regular aging process: (i) the reduction of regenerative ability of the olfactory mucosa; (ii) the decreasing of olfactory receptor neurons (Conley et al., 2003; Suzukawa et al., 2011); (iii) the slowing down of cell turnover in the olfactory epithelium (Conley et al., 2003).

Birte-Antina et al. (2018) have found that despite the loss of olfactory function is largely found with aging, discontinuous exposure to odors improves general olfactory function. Olfactory training in older people improved general olfactory function, verbal function, subjective well-being, and, in a subgroup, also depressive symptoms. Kondo et al. (2020) remarked that the mechanism underlying odor stimulation-dependent improvement is unclear but the number of interneurons in the olfactory bulb is regulated depending on odor stimulation (Yokoyama et al., 2011).

Therefore, although the protocol proposed follows the main guidelines of the protocols founded in the literature, our target sample is older than the previously studied and, the training protocol proposed presents possible improvements in terms of simplicity, follow-up and adherence comparing to the existent evidence. In addition, the expected sensorial improvement will be accompanied by unprecedented personalized OE proteotypes (composed around quantitative data from 4500 to 5000 proteins) that will help us to understand the unknown molecular mechanisms triggered by the OT. Moreover, this workflow has the capacity to unveil protein mediators that may be targeted by future therapies in order to improve the odor capacity and the olfactory recovery.

Research in the field of immunology, as well as in various neurological disorders, indicate the increasing relevance of smell. In this study, we pretend to implement olfactory precision medicine through immunodulatory nasal therapies, so the results obtained from immune populations, before and after OT, could shed a light on the brain-smell-immune system axis.

To sum-up our hypothesis indicates that the structured, short-term exposure to odors may increase olfactory sensitivity (Hummel et al., 2009). However, the magnitude of this effect and its causality are still unknown in the elderly population. Following the literature, the main management strategies for age-related olfactory dysfunction are odor stimulation (Yokoyama et al., 2011; Kondo et al., 2020), OT (Schriever et al., 2014) and exercise regularly (Schubert et al., 2013).

## Trial Status

The Hospital Universitario de Navarra Clinical Research Ethics Committee approved the protocol on October 21, 2020 (PI_2020/113). Regarding molecular analysis, cell cultures, protein extraction, precipitation, digestion, and peptide desalting/cleaning protocols are optimized to analyze human OE. Preliminar mass-spectrometry analysis have allowed to generate a first draft of the human OE proteome, identifying 4500 proteins using a 5600 triple-TOF mass-spectrometer (ABSciex). This dataset has allowed us to optimize different bioinformatic tools and pathway databases to extract functional information from protein-centric olfactory studies.

## Ethics Statement

This study follow the principles of the Declaration of Helsinki (World Medical Association) and was approved by the Hospital Universitario de Navarra Clinical Research Ethics Committee on October 21, 2020 (PI_2020/113). All volunteers or their legal representatives will be asked to sign a written informed consent form, also approved in advance by the Ethics Committee.

## Author Contributions

FíZ-F, ML-M, NC, and AG developed and drafted the study protocol in consultation with FrZ-F, NM-V, ES, JF-I, JL, PC-C, SF, BC-V, EM-A, and MU-L. JF-I, ES, FrZ-F, and NM-V prepared the human research ethics. NM-V, FíZ-F, FrZ-F, BC-V, EM-A, and MU-L involved in the facilitation of the group program intervention. JF-I, ES, NC, and AG involved in the mixed-methods research methodologies. All authors listed were involved in the overall research design and selection of outcome measures, have made a substantial, direct and intellectual contribution to the work, and approved it for publication.

## Conflict of Interest

The authors declare that the research was conducted in the absence of any commercial or financial relationships that could be construed as a potential conflict of interest.

## Publisher’s Note

All claims expressed in this article are solely those of the authors and do not necessarily represent those of their affiliated organizations, or those of the publisher, the editors and the reviewers. Any product that may be evaluated in this article, or claim that may be made by its manufacturer, is not guaranteed or endorsed by the publisher.

## References

[B1] Abolmaali N. D. Hietschold V. Vogl T. J. Huttenbrink K. B. Hummel T. (2002). MR evaluation in patients with isolated anosmia since birth or early childhood. *Am. J. Neuroradiol.* 23 157–164. 11827889 PMC7975503

[B2] Al Aïn S. Poupon D. Hétu S. Mercier N. Steffener J. Frasnelli J. (2019). Smell training improves olfactory function and alters brain structure. *Neuroimage* 189 45–54. 10.1016/j.neuroimage.2019.01.008 30630079

[B3] Amoore J. E. (1977). Specific anosmia and the concept of primary odors. *Chem. Senses* 2 267–281. 10.1093/chemse/2.3.267

[B4] Badia X. Roset M. Montserrat S. Herdman M. Segura A. (1999). The Spanish version of EuroQol: a description and its applications. European Quality of Life scale. *Med. Clin.* 112 79–85. 10618804

[B5] Barrera-Conde M. Ausin K. Lachén-Montes M. Fernández-Irigoyen J. Galindo L. Cuenca-Royo A. (2021). Cannabis use induces distinctive proteomic alterations in olfactory neuroepithelial cells of schizophrenia patients. *J. Pers. Med.* 11:160. 10.3390/jpm11030160 33668817 PMC7996288

[B6] Birte-Antina W. Ilona C. Antje H. Thomas H. (2018). Olfactory training with older people. *Int. J. Geriatr. Psychiatry* 33 212–220. 10.1002/gps.4725 28429377

[B7] Cancela J. M. Ayán C. Varela S. (2012). “Symbol Digit Modalities Test” normative values for Spanish home care residents: a pilot study. *Actas Espanolas Psiquiatr.* 40 299–303. 23165411

[B8] Conley D. B. Robinson A. M. Shinners M. J. Kern R. C. (2003). Age-related olfactory dysfunction: cellular and molecular characterization in the rat. *Am. J. Rhinol.* 17 169–175. 10.1177/194589240301700311 12862407

[B9] Dalton P. Doolittle N. Breslin P. A. S. (2002). Gender-specific induction of enhanced sensitivity to odors. *Nat. Neurosci.* 5 199–202. 10.1038/nn803 11865309

[B10] Damm M. Pikart L. K. Reimann H. Burkert S. Goktas O. Haxel B. (2014). Olfactory training is helpful in postinfectious olfactory loss - a randomized controlled multicenter study. *Laryngoscope* 124 826–831. 10.1002/lary.24340 23929687

[B11] Delgado-Losada M. L. Delgado-Lima A. H. Bouhaben J. (2020). Spanish validation for olfactory function testing using the sniffin’sticks olfactory test: threshold, discrimination, and identification. *Brain Sci.* 10:943. 10.3390/brainsci10120943 33297359 PMC7762307

[B12] Deuschl G. Beghi E. Fazekas F. Varga T. Christoforidi K. A. Sipido E. (2020). The burden of neurological diseases in Europe: an analysis for the Global Burden of Disease Study 2017. *Lancet Public Health* 5 e551–e567. 10.1016/S2468-2667(20)30190-0 33007212

[B13] Djordjevic J. Jones-Gotman M. De Sousa K. Chertkow H. (2008). Olfaction in patients with mild cognitive impairment and Alzheimer’s disease. *Neurobiol. Aging* 29 693–706. 10.1016/j.neurobiolaging.2006.11.014 17207898

[B14] Doty R. L. (2017). Olfactory dysfunction in neurodegenerative diseases: is there a common pathological substrate? *Lancet Neurol.* 16 478–488. 10.1016/S1474-4422(17)30123-0 28504111

[B15] Doty R. L. (2019). Epidemiology of smell and taste dysfunction. *Handb. Clin. Neurol.* 164 3–13. 10.1016/B978-0-444-63855-7.00001-0 31604555

[B16] Doty R. L. Petersen I. Mensah N. Christensen K. (2011). Genetic and environmental influences on odor identification ability in the very old. *Psychol. Aging* 26, 864–871. 10.1037/a0023263 21639645 PMC3631779

[B17] Doty R. L. Shaman P. Applebaum S. L. Giberson R. Siksorski L. Rosenberg L. (1984). Smell identification ability: changes with age. *Science* 226, 1441–1443. 10.1126/science.6505700 6505700

[B18] Doty R. L. Snyder P. J. Huggins G. R. Lowry L. D. (1981). Endocrine, cardiovascular, and psychological correlated of olfactory sensitivity changes during the human menstrual cycle. *J. Comp. Physiol. Psychol.* 95 45–60. 10.1037/h0077755 6783690

[B19] Engen T. (1960). Effect of practice and instruction on olfactory thresholds. *Percept. Mot. Skills* 10 195–198. 10.2466/pms.1960.10.3.195

[B20] Fjaeldstad A. W. Ovesen T. Hummel T. (2021). The association between smoking on olfactory dysfunction in 3,900 patients with olfactory loss. *Laryngoscope* 131 E8–E13. 10.1002/lary.28552 32096874

[B21] Fleiner F. Lau L. Göktas Ö (2012). Active olfactory training for the treatment of smelling disorders. *Ear Nose Throat. J.* 91 198–203. 10.1177/014556131209100508 22614554

[B22] Folstein M. F. Folstein S. E. McHugh P. R. (1975). “Mini-mental state”: a practical method for grading the cognitive state of patients for the clinician. *J. Psychiatr. Res.* 12, 189–198. 1202204 10.1016/0022-3956(75)90026-6

[B23] Fornazieri M. A. Garcia E. C. D. Lopes N. M. D. Miyazawa I. N. I. Silva G. dos S. (2019). Adherence and efficacy of olfactory training as a treatment for persistent olfactory loss. *Am. J. Rhinol. Allergy* 34 238–248. 10.1177/1945892419887895 31766853

[B24] García-Gordillo M. Á del Pozo-Cruz B. Adsuar J. C. Cordero-Ferrera J. M. Abellán-Perpiñán J. M. Sánchez-Martínez F. I (2015). Validation and comparison of EQ-5D-3L and SF-6D instruments in a Spanish Parkinson’s disease population sample. *Nutr. Hosp.* 32 2808–2821. 26667738 10.3305/nh.2015.32.6.9765

[B25] Gudziol V. Lotsch J. Hahner A. Zahnert T. Hummel T. (2006). Clinical significance of results from olfactory testing. *Laryngoscope* 116 1858–1863. 10.1097/01.mlg.0000234915.51189.cb 17003712

[B26] Guralnik J. M. Simonsick E. M. Ferrucci L. Glynn R. J. Berkman L. F. Blazer D. G. (1994). A short physical performance battery assessing lower extremity function: association with self-reported disability and prediction of mortality and nursing home admission. *J. Gerontol.* 49 M85–M94. 10.1093/geronj/49.2.M85 8126356

[B27] Hadley K. Orlandi R. R. Fong K. J. (2004). Basic anatomy and physiology of olfaction and taste. *Otolaryngol. Clin. N.A.* 37 1115–1126. 10.1016/j.otc.2004.06.009 15563905

[B28] Haehner A. Tosch C. Wolz M. Klingelhoefer L. Fauser M. Storch A. (2013). Olfactory training in patients with Parkinson’s disease. *PLoS One* 8:e61680. 10.1371/journal.pone.0061680 23613901 PMC3629137

[B29] Hummel T. (2014). Olfaction and olfactory disorders. *HNO* 62 845–845. 10.1007/s00106-014-2939-9 25323732

[B30] Hummel T. Kobal G. Gudziol H. Mackay-Sim A. (2007). Normative data for the “Sniffin’ Sticks” including tests of odor identification, odor discrimination, and olfactory thresholds: an upgrade based on a group of more than 3,000 subjects. *Eur. Arch. Otorhinolaryngol.* 264 237–243. 10.1007/s00405-006-0173-0 17021776

[B31] Hummel T. Rissom K. Reden J. Hahner A. Weidenbecher M. Huttenbrink K. B. (2009). Effects of olfactory training in patients with olfactory loss. *Laryngoscope* 119 496–499. 10.1002/lary.20101 19235739

[B32] Hummel T. Sekinger B. Wolf S. R. Pauli E. Kobal G. (1997). ‘Sniffin’sticks’: olfactory performance assessed by the combined testing of odor identification, odor discrimination and olfactory threshold. *Chem. Senses* 22 39–52. 10.1093/chemse/22.1.39 9056084

[B33] Knudsen K. Flensborg Damholdt M. Mouridsen K. Borghammer P. (2015). Olfactory function in Parkinson’s Disease–effects of training. *Acta Neurol. Scand.* 132 395–400. 10.1111/ane.12406 25846906

[B34] Kondo K. Kikuta S. Ueha R. Suzukawa K. Yamasoba T. (2020). Age-related olfactory dysfunction: epidemiology, pathophysiology, and clinical management. *Front. Aging Neurosci.* 12:208. 10.3389/fnagi.2020.00208 32733233 PMC7358644

[B35] Konstantinidis I. Tsakiropoulou E. Bekiaridou P. Kazantzidou C. Constantinidis J. (2013). Use of olfactory training in post-traumatic and postinfectious olfactory dysfunction. *Laryngoscope* 123 E85–E90. 10.1002/lary.24390 24114690

[B36] Lachén-Montes M. Fernández-Irigoyen J. Santamaría E. (2016). Deconstructing the molecular architecture of olfactory areas using proteomics. *Proteomics Clin. Appl.* 10 1178–1190. 10.1002/prca.201500147 27226001

[B37] Lachén-Montes M. González-Morales A. Iloro I. Elortza F. Ferrer I. Gveric D. (2019a). Unveiling the olfactory proteostatic disarrangement in Parkinson’s disease by proteome-wide profiling. *Neurobiol. Aging* 73 123–134. 10.1016/j.neurobiolaging.2018.09.018 30342273

[B38] Lachén-Montes M. González-Morales A. Schvartz D. Zelaya M. V. Ausin K. Fernández-Irigoyen J. (2019b). The olfactory bulb proteotype differs across frontotemporal dementia spectrum. *J. Proteom.* 201 37–47. 10.1016/j.jprot.2019.04.011 30999060

[B39] Lachén-Montes M. González-Morales A. Zelaya M. V. Pérez-Valderrama E. Ausín K. Ferrer I. (2017). Olfactory bulb neuroproteomics reveals a chronological perturbation of survival routes and a disruption of prohibitin complex during Alzheimer’s disease progression. *Sci. Rep.* 7:9115. 10.1038/s41598-017-09481-x 28831118 PMC5567385

[B40] Lachén-Montes M. Mendizuri N. Ausin K. Andrés-Benito P. Ferrer I. Fernández-Irigoyen J. (2020a). Amyotrophic lateral sclerosis is accompanied by protein derangements in the olfactory bulb-tract axis. *Int. J. Mol. Sci.* 21:8311. 10.3390/ijms21218311 33167591 PMC7664257

[B41] Lachén-Montes M. Mendizuri N. Ausín K. Peìrez-Mediavilla A. Azkargorta M. Iloro I. (2020b). Smelling the dark proteome: functional characterization of PITH domain-containing protein 1 (C1orf128) in olfactory metabolism. *J. Proteome Res.* 19 4826–4843. 10.1021/acs.jproteome.0c00452 33185454

[B42] Lasarte-Cia A. Lozano T. Pérez-González M. Gorraiz M. Iribarren K. Hervás-Stubbs S. (2018). Immunomodulatory properties of carvone inhalation and its effects on contextual fear memory in mice. *Front. Immunol.* 9:68. 10.3389/fimmu.2018.00068 29422905 PMC5788902

[B43] Mahoney F. I. Barthel D. W. (1965). Functional evaluation: the Barthel Index: a simple index of independence useful in scoring improvement in the rehabilitation of the chronically ill. *Md State Med. J.* 14 61–65. 14258950

[B44] Marin C. Vilas D. Langdon C. Alobid I. López-Chacón M. Haehner A. (2018). Olfactory dysfunction in neurodegenerative diseases. *Curr. Allergy Asthma Rep.* 18:42. 10.1007/s11882-018-0796-4 29904888

[B45] Martínez J. Onís M. C. Dueñas R. Albert C. Aguado C. Luque R. (2002). Versión española del cuestionario de Yesavage abreviado (GDS) para el despistaje de depresión en mayores de 65 años: adaptación y validación. *Medifam* 12 620–630. 10.4321/S1131-57682002001000003

[B46] Masala C. Saba L. Cecchini M. P. Solla P. Loy F. (2018). Olfactory function and age: a Sniffin’ sticks extended test study performed in Sardinia. *Chem. Percept.* 11, 19–26. 10.1007/s12078-017-9233-7

[B47] McGrath R. P. Kraemer W. J. Al Snih S. Peterson M. D. (2018). Handgrip strength and health in aging adults. *Sports Med.* 48 1993–2000. 10.1007/s40279-018-0952-y 29943230

[B48] Melis M. Sollai G. Masala C. Pisanu C. Cossu G. Melis M. (2019). Odor identification performance in idiopathic Parkinson’s disease is associated with gender and the genetic variability of the olfactory binding protein. *Chem. Senses* 44, 311–318. 10.1093/chemse/bjz020 30944919

[B49] Moein S. T. Hashemian S. M. Mansourafshar B. Khorram-Tousi A. Tabarsi P. Doty R. L. (2020). Smell dysfunction: a biomarker for COVID-19. *Int. Forum Allergy Rhinol.* 10, 944–950. 10.1002/alr.22587 32301284 PMC7262123

[B50] Mueller A. Rodewald A. Reden J. Gerber J. Von Kummer R. Hummel T. (2005). Reduced olfactory bulb volume in post-traumatic and post-infectious olfactory dysfunction. *Neuroreport* 16 475–478. 10.1097/00001756-200504040-00011 15770154

[B51] Mullol J. Alobid I. Mariño-Sánchez F. Quintó L. de Haro J. Bernal-Sprekelsen M. (2012). Furthering the understanding of olfaction, prevalence of loss of smell and risk factors: a population-based survey (OLFACAT study). *BMJ Open* 2:e001256. 10.1136/bmjopen-2012-001256 23135536 PMC3533119

[B52] Murphy C. Schubert C. R. Cruickshanks K. J. Klein B. E. Klein R. Nondahl D. M. (2002). Prevalence of olfactory impairment in older adults. *JAMA* 288 2307–2312. 10.1001/jama.288.18.2307 12425708

[B53] Ostrosky-Solís F. López-Arango G. Ardila A. (2000). Sensitivity and specificity of the mini-mental state examination in a spanish-speaking population. *Appl. Neuropsychol.* 7 25–31. 10.1207/S15324826AN0701_4 10800625

[B54] P.R.O-V1.1E (2020). *Olfactory Reeducation Protocol – Spanish Version.* Available online at: https://asociacionanosmia.com/wp-content/uploads/2021/02/Protocolo-de-reeducacion-olfativa-pro-v1-1s.pdf (accessed July 20, 2021).

[B55] Patel Z. M. (2016). The evidence for olfactory training in treating patients with olfactory loss. *Curr. Opin. Otolaryngol. Head Neck Surg.* 25 43–46. 10.1097/moo.0000000000000328 27841770

[B56] Pekala K. Chandra R. K. Turner J. H. (2016). Efficacy of olfactory training in patients with olfactory loss: a systematic review and meta-analysis. *Int. Forum Allergy Rhinol.* 6 299–307. 10.1002/alr.21669 26624966 PMC4783272

[B57] Perera S. Mody S. H. Woodman R. C. Studenski S. A. (2006). Meaningful change and responsiveness in common physical performance measures in older adults. *J. Am. Geriatr. Soc.* 54 743–749. 10.1111/j.1532-5415.2006.00701.x 16696738

[B58] Pi J. Olivé J. M. Esteban M. (1994). Mini Mental State Examination: association of the score obtained with the age and degree of literacy in an aged population. *Med. Clin.* 103 641–644. 7808061

[B59] Rabin M. D. Cain W. S. (1986). Determinants of measured olfactory sensitivity. *Percept. Psychol. Phys.* 39 281–286. 10.3758/BF03204936 3737357

[B60] Rantanen T. Guralnik J. M. Foley D. Masaki K. Leveille S. Curb J. D. (1999). Midlife hand grip strength as a predictor of old age disability. *JAMA* 281 558–560. 10.1001/jama.281.6.558 10022113

[B61] Reyes-de-Beaman S. Beaman P. E. Garcia-Peña C. Villa M. A. Heres J. Córdova A. (2004). Validation of a modified version of the mini-mental state examination (MMSE) in Spanish. *Aging Neuropsychol. Cogn.* 11 1–11. 10.1076/anec.11.1.1.29366

[B62] Rombaux P. Weitz H. Mouraux A. Nicolas G. Bertrand B. Duprez T. (2006). Olfactory function assessed with orthonasal and retronasal testing, olfactory bulb volume, and chemosensory event-related potentials. *Arch. Otolaryngol. Head Neck Surg.* 132 1346–1351. 10.1001/archotol.132.12.1346 17178947

[B63] Rumeau C. Nguyen D. T. Jankowski R. (2016). How to assess olfactory performance with the Sniffin’Sticks test^®^. *Eur. Ann. Otorhinolaryngol. Head Neck Dis.* 133 203–206. 10.1016/j.anorl.2015.08.004 26344139

[B64] Sanna F. Loy F. Piras R. Moat A. Masala C. (2021). Age-related cognitive decline and the olfactory identification deficit are associated to increased level of depression. *Front. Neurosci.* 15:599593. 10.3389/fnins.2021.599593 33692667 PMC7937898

[B65] Schiffman S. S. (1997). Taste and smell losses in normal aging and disease. *JAMA* 278 1357–1362. 10.1001/jama.1997.03550160077042 9343468

[B66] Schriever V. A. Lehmann S. Prange J. Hummel T. (2014). Preventing olfactory deterioration: olfactory training may be of help in older people. *J. Am. Geriatr. Soc.* 62 384–386. 10.1111/jgs.12669 24521370

[B67] Schubert C. R. Cruickshanks K. J. Nondahl D. M. Klein B. E. Klein R. Fischer M. E. (2013). Association of exercise with lower long-term risk of olfactory impairment in older adults. *JAMA Otolaryngol. Head Neck Surg.* 139 1061–1066. 10.1001/jamaoto.2013.4759 24135745 PMC3855446

[B68] Sedaghat A. R. Gengler I. Speth M. M. (2020). Olfactory dysfunction: a highly prevalent symptom of COVID-19 with public health significance. *Otolaryngol. Head Neck Surg.* 163 12–15. 10.1177/0194599820926464 32366160

[B69] Shah S. Vanclay F. Cooper B. (1989). Improving the sensitivity of the Barthel Index for stroke rehabilitation. *J. Clin. Epidemiol.* 42 703–709. 10.1016/0895-4356(89)90065-6 2760661

[B70] Smith A. (1973). *Symbol Digit Modalities Test.* Los Angeles, CA: Western Psychological Services.

[B71] Smith A. (2002). *SDMT: Test De Símbolos Y Dígitos: Manual.* Madrid: TEA Ediciones, 2002.

[B72] Solla P. Masala C. Liscia A. Piras R. Ercoli T. Fadda L. (2020). Sex-related differences in olfactory function and evaluation of possible confounding factors among patients with Parkinson’s disease. *J. Neurol.* 267, 57–63. 10.1007/s00415-019-09551-2 31555978

[B73] Sorokowska A. Schriever V. A. Gudziol V. Hummel C. Hähner A. Iannilli E. (2015). Changes of olfactory abilities in relation to age: odor identification in more than 1400 people aged 4 to 80 years. *Eur. Arch. Otorhinolaryngol.* 272 1937–1944. 10.1007/s00405-014-3263-4 25238811 PMC4473282

[B74] Sorokowski P. Karwowski M. Misiak M. Marczak M. K. Dziekan M. Hummel T. (2019). Sex differences in human olfaction: a meta-analysis. *Front. Psychol.* 10:242. 10.3389/fpsyg.2019.00242 30814965 PMC6381007

[B75] Strous R. D. Shoenfeld Y. (2006). To smell the immune system: olfaction, autoimmunity and brain involvement. *Autoimmun. Rev.* 6 54–60. 10.1016/j.autrev.2006.07.002 17110318

[B76] Suzukawa K. Kondo K. Kanaya K. Sakamoto T. Watanabe K. Ushio M. (2011). Age-related changes of the regeneration mode in the mouse peripheral olfactory system following olfactotoxic drug methimazole-induced damage. *J. Comp. Neurol.* 519 2154–2174. 10.1002/cne.22611 21452219

[B77] Tombaugh T. N. (2004). Trail Making Test A and B: normative data stratified by age and education. *Arch. Clin. Neuropsychol.* 19 203–214. 10.1016/S0887-6177(03)00039-8 15010086

[B78] Turner J. H. (2020). Olfactory training: what is the evidence? *Int. Forum Allergy Rhinol.* 10 1199–1200. 10.1002/alr.22681 32776673 PMC7668391

[B79] United Nations (2019). *World Population Prospects 2019.* Available online at: https://population.un.org/wpp/DataQuery/ (accessed July 20, 2021).

[B80] van Bennekom C. A. Jelles F. Lankhorst G. J. Bouter L. M. (1996). Responsiveness of the rehabilitation activities profile and the Barthel Index. *J. Clin. Epidemiol.* 49 39–44. 10.1016/0895-4356(95)00559-5 8598509

[B81] van Dongen J. J. Van Der Burg M. Kalina T. Perez-Andres M. Mejstrikova E. Vlkova M. (2019). EuroFlow-based flowcytometric diagnostic screening and classification of primary immunodeficiencies of the lymphoid system. *Front. Immunol.* 10:1271. 10.3389/fimmu.2019.01271 31263462 PMC6585843

[B82] Vennemann M. M. Hummel T. Berger K. (2008). The association between smoking and smell and taste impairment in the general population. *J. Neurol.* 255 1121–1126. 10.1007/s00415-008-0807-9 18677645

[B83] World Medical Association (2013). World Medical Association Declaration of Helsinki: ethical principles for medical research involving human subjects. *JAMA* 310 2191–2194. 10.1001/jama.2013.281053 24141714

[B84] Yokoyama T. K. Mochimaru D. Murata K. Manabe H. Kobayakawa K. Kobayakawa R. (2011). Elimination of adult-born neurons in the olfactory bulb is promoted during the postprandial period. *Neuron* 71 883–897. 10.1016/j.neuron.2011.05.046 21903081

